# Variation in the efficiency of English general practices and associated factors: A cross-sectional study of 5069 general practices

**DOI:** 10.1080/13814788.2025.2580827

**Published:** 2025-11-17

**Authors:** Augustine Onwunduba, Jianhua Wu, Helena Painter, Helen Pearce, John Ford

**Affiliations:** a Centre for Primary Care, Wolfson Institute of Population Health, Queen Mary University of London, London, UK; b Department of Clinical Pharmacy and Pharmacy Management, Nnamdi Azikiwe University, Awka, Nigeria

**Keywords:** Primary care, general practice, efficiency, machine learning

## Abstract

**Background:**

Healthcare demand in English general practice exceeds supply, necessitating practice efficiency. To our knowledge, no study has explored factors associated with practice efficiency in England using a quality-adjusted output.

**Objectives:**

To determine practice-level efficiency in England and identify associated factors using a quality-adjusted output.

**Methods:**

We conducted a cross-sectional study using NHS datasets from 2023. Practice efficiency was determined by comparing input (measured using funding and workforce) with output (measured using clinical quality, patient experience, and service volume). Practices were classified as efficient (low input, high output), neutral (same input and output levels), or inefficient (high input, low output) using K-medoids clustering, a machine learning technique. Multivariable logistic regression was used to identify factors associated with practice efficiency (i.e. efficient or inefficient, excluding neutral).

**Results:**

Of 5069 practices, 1117 were classified as efficient, 2773 as neutral, and 1179 as inefficient. Efficiency was lower in practices with a larger patient list (adjusted odds ratio 0.23, 95% CI 0.19–0.28), those with a higher percentage of patients with a chronic condition (0.47, 0.38–0.58) or patients ≥ 65 years (0.63, 0.49–0.81), those in a higher deprivation area (0.25, 0.20–0.32), those that dispense medications (0.52, 0.37–0.73), and those with an alternative provider medical services (vs. general medical services) contract (0.15, 0.07–0.33). Efficiency was higher in urban practices (1.38, 1.00–1.90) and practices with a higher percentage of mixed (1.66, 1.24–2.21) or other ethnicity patients (1.78, 1.22–2.60).

**Conclusion:**

Smaller practices were more efficient. Therefore, policies that encourage practice mergers may not deliver the efficiency gains expected.

## KEY MESSAGE

In England, smaller general practices tend to be more efficient, optimising input and output more effectively than larger general practices.

Policymakers should not assume that actions, such as general practice mergers, which typically generate economies of scale, will necessarily improve general practice efficiency.

## Introduction

Previous research has shown that healthcare demand in English general practice exceeds supply due to a growing and ageing population [1].

Recruiting more general practitioners (GPs) could help address this imbalance, but it requires time and investment [2]. In the interim, it is important for general practices to operate efficiently. Primary care efficiency has been defined in different ways, including in terms of the interval between a patient’s consultation and their next one [3] and in terms of input and output. Here, we adopt the latter definition, which is how efficiency is defined traditionally. An efficient general practice delivers high output (typically measured using volume of services or quality of care) with low input (measured using attributes such as funding level and workforce capacity), while an inefficient general practice delivers low output with high input. Research has found that efficiency in English general practice is declining, with an estimated fall of 0.1%, on average, annually between 2012/2013 and 2019/2020 [2]. Current policy efforts to improve English general practice efficiency include expanding multidisciplinary teams, such as advanced nurse practitioners, physician associates, and pharmacists [4]; allowing community pharmacies to take on more clinical roles [5]; using online consultations [6]; and merging general practices [7].

A better understanding of factors contributing to general practice efficiency is needed to optimise policy. Previous studies, conducted over the last three decades, have reported factors associated with primary care efficiency. In general, primary care organisations that serve populations with lower health needs and those that do not dispense medications are more efficient [8–13].

Unfortunately, none of the previous studies determined primary care efficiency using a quality-adjusted output, defined in terms of both quality of care (measured, at a minimum, using clinical quality and patient experience – consistent with The King’s Fund’s identification of these as key domains of healthcare quality that should be considered jointly to provide a comprehensive picture of quality of care [14]) and volume of services (measured using attributes such as volume of consultations). They mainly defined output in terms of volume of services or quality of care, but not both. A quality-adjusted output is important because performance with respect to a combination of throughput and quality of care is arguably more important than performance with respect to either alone.

Accordingly, we aimed to use a quality-adjusted output to identify factors associated with general practice-level efficiency in England in 2023. The specific objectives were: (1) to identify general practices that were efficient, neutral, or inefficient in 2023, and (2) to identify factors associated with general practice efficiency (i.e. efficient or inefficient, excluding neutral) in 2023.

## Methods

### Study design and data sources

This was a cross-sectional study using the following NHS datasets: General Practice Workforce [15], General Practice Patient Survey [16], NHS Payments to General Practice [17], Quality and Outcomes Framework (QOF) [18], National General Practice Profiles [19], Appointments in General Practice [20], and Ethnicity [21].

The NHS directly generates some of the data (e.g. general practice funding data). It obtains the others from patients (e.g. patient experience data), general practices (e.g. workforce data), or other sources (e.g. deprivation data). The data are from 2023, except deprivation data, which was released in 2019.

### Participants

The study population comprised English general practices. We excluded general practices that met any of the following criteria:

- Missing or invalid data.
- More than 10 GPs per 10,000 patients or fewer than 1 GP per 10,000 patients.
- More than 10 nurses per 10,000 patients or fewer than 0.5 nurses per 10,000 patients.
- More than 10 direct patient care (DPC) staff per 10,000 patients.
- More than 30 administrative staff per 10,000 patients.
- General practice ID not consistently available across all the years considered in our longitudinal analysis of general practice efficiency (the findings of which we will disseminate separately).

### Factors

Several factors (variables) were studied to explore their impact on general practice efficiency. These include: number of patients (high vs. low); rurality (urban vs. rural); dispensing (dispensing vs. non-dispensing); contract type (alternative provider medical services vs. general medical services, and personal medical services vs. general medical services); ethnicity of patients, including white (high vs. low), black (high vs. low), mixed (high vs. low), Asian (high vs. low), and other (high vs. low); chronic conditions (high vs. low); deprivation score (high vs. low); and patients 65 years or older (high vs. low). Although we explored their associations with the outcome, the ethnicity factors were primarily selected to serve as covariates. The other factors listed above were informed by the English primary care efficiency literature [8–13]. Table 1 provides further information about all factors.

**Table 1.** Factors studied, their definitions, and their sources.

Factor		Definition	Source NHS dataset
*Number of patients		*Number of patients* categorises a general practice as ‘high’ or ‘low’ based on the number of patients it has.	General Practice Workforce
Rurality		*Rurality* categorises a general practice as ‘rural’ or ‘urban’ based on its geographical location.	NHS Payments to General Practice
Dispensing		*Dispensing* categorises a general practice as ‘dispensing’ or ‘non-dispensing’ based on whether or not it dispenses medications.	
Contract type		*Contract type* categorises a general practice as ‘PMS’, ‘GMS’ or ‘APMS’ based on the contract type it has.	
*Chronic conditions		*Chronic conditions* categorises a general practice as ‘high’ or ‘low’ based on the percentage of patients – with a chronic condition – it has.	National General Practice Profiles
*Patients 65 years or older		*Patients 65 years or older* categorises a general practice as ‘high’ or ‘low’ based on the percentage of patients – aged 65 years or older – it has.	
*Deprivation score		*Deprivation score* categorises a general practice as ‘high’ or ‘low’ based on its deprivation score (2019) as determined by its geographical location. The deprivation score tells us how deprived an area in England is, with respect to factors like employment, income, etc. The higher the score, the more deprived an area is.	
*Ethnicity	White	*White* categorises a general practice as ‘high’ or ‘low’ based on the percentage of white patients it has. General practices with a higher percentage were classified as ‘high’. Those with a lower percentage were classified as ‘low’.	Ethnicity
	Black	*Black* categorises a general practice as ‘high’ or ‘low’ based on the percentage of black patients it has.	
	Mixed	*Mixed* categorises a general practice as ‘high’ or ‘low’ based on the percentage of mixed patients it has. *Mixed* patients are those whose parents are from different ethnic backgrounds (e.g. one white parent and one black parent).	
	Asian	*Asian* categorises a general practice as ‘high’ or ‘low’ based on the percentage of Asian patients it has.	
	Other	*Other* categorises a general practice as ‘high’ or ‘low’ based on the percentage of patients – with their ethnicity defined as *Other* – it has. *Other* patients are those who are not categorised as white, black, mixed, or Asian.	

* Denotes a variable that came from the NHS as a numerical variable but was subsequently converted to a categorical variable with two categories – high and low – using K-medoids clustering.

APMS: Alternative Provider Medical Services; GMS: General Medical Services; PMS: Personal Medical Services. In England, GMS, PMS, and APMS are the three types of contract between the NHS and general practices. Among other things, each contract specifies the primary care services that can be provided by general practices that hold it. GMS, the standard contract, is held by most general practices, and it enables them to provide core services. PMS is held by some general practices, and it allows them to provide core services. PMS makes it possible to tailor services to local needs. APMS is held by very few general practices, and it permits them to provide core or non-core services.

### Outcome

The outcome was the efficiency status of a general practice (i.e. efficient or inefficient).

#### Efficiency determination

The efficiency of a general practice was determined using its input and output. Input was defined by the following variables: general practice funding, GP number, nurse number, DPC staff number, and administrative staff number. Output was defined by the following variables: access, confidence, continuity, overall experience, QOF regarding hypertension, QOF regarding diabetes, cancer detection, and general practice appointments. The input and output variables are defined in Tables 2 and 3, respectively. These variables were informed by the primary care efficiency literature [22].

**Table 2.** Input variables, their definitions, and their sources.

Input variable	Definition	Source NHS dataset
*GP number	*GP number* represents the full-time equivalent of the hours worked by a general practice’s GPs (full-time and locum GPs) per 10,000 patients.	General Practice Workforce
*Nurse number	*Nurse number* represents the full-time equivalent of the hours worked by a general practice’s nurses per 10,000 patients.	
*DPC staff number	*DPC staff number* represents the full-time equivalent of the hours worked by a general practice’s direct patient care staff per 10,000 patients.	
*Administrative staff number	*Administrative staff number* represents the full-time equivalent of the hours worked by a general practice’s administrative staff per 10,000 patients.	
General practice funding	*General practice funding* represents the total NHS payment in £ (including COVID and PCN payments and excluding deductions and QOF-related payments) to a general practice per 10,000 patients.	NHS Payments to General Practice

* Data are a snapshot as of 31 December 2023.

DPC: Direct Patient Care; GP: General Practitioner; PCN: Primary Care Network; QOF: Quality and Outcomes Framework.

DPC staff are staff, other than nurses or GPs, directly involved in patient care.

**Table 3.** Output variables, their definitions, and their sources.

Variable	Definition	Source NHS dataset
Access	*Access* represents the percentage of a general practice’s patients who reported ‘very good’ or ‘fairly good’ when asked to describe their overall experience of making an appointment. They were provided with the following possible responses: ‘very good’, ‘fairly good’, ‘neither good nor poor’, ‘fairly poor’, and ‘very poor’.	General Practice Patient Survey
Confidence	*Confidence* represents the percentage (excluding ‘don’t know’ or ‘can’t say’ from the base) of a general practice’s patients who reported ‘yes, definitely’ or ‘yes, to some extent’ when asked whether they had confidence and trust in the health care professional who attended to them during their last general practice appointment. They were provided with the following possible responses: ‘yes, definitely’, ‘yes, to some extent’, ‘no, not at all’, and ‘don’t know’ or ‘can’t say’.	
Continuity	*Continuity* represents the percentage (excluding ‘I have not tried’ from the base) of a general practice’s patients who reported ‘always or almost always’ or ‘a lot of the time’ when asked the frequency with which they see or speak to their preferred general practitioner. They were provided with the following possible responses: ‘always or almost always’, ‘a lot of the time’, ‘some of the time’, ‘never or almost never’, and ‘I have not tried’.	
Overall experience	*Overall experience* represents the percentage of a general practice’s patients who reported ‘very good’ or ‘fairly good’ when asked to describe their overall experience of their general practice. They were provided with the following possible responses: ‘very good’, ‘fairly good’, ‘neither good nor poor’, ‘fairly poor’, and ‘very poor’.	
QOF regarding hypertension	*QOF regarding hypertension* represents the percentage of a general practice’s hypertensive patients aged 79 years or younger with a reading of 140/90 mmHg or less in their last blood pressure measurement taken in the preceding 12 months.	Quality and Outcomes Framework
QOF regarding diabetes	*QOF regarding diabetes* represents the percentage of a general practice’s diabetic patients – without severe or moderate frailty – with a reading of 58 mmol/mol or less in their last IFCC-HbA1c test done in the preceding 12 months.	
Cancer detection	*Cancer detection* represents the percentage of a general practice’s newly diagnosed and treated cancer patients who were first diagnosed after the general practice suspected they had cancer and referred them for further evaluation via the two-week wait referral pathway.	National General Practice Profiles
General practice appointments	*General practice appointments* represents the number of appointments made by a general practice per 10,000 patients in December 2023.	Appointments in General Practice

QOF: Quality and Outcomes Framework.

QOF is a system used in the UK to incentivise general practices to provide high-quality care to patients. The QOF system has indicators (e.g. QOF regarding hypertension), and general practices are awarded QOF points based on how well they perform against the QOF indicators.

Effectively, data on the input and output variables, across different NHS datasets, were linked using general practice ID to create a linked data. K-medoids clustering was then applied to the input variables in the linked data to classify general practices as having ‘high’ or ‘low’ input, based on the size of the medoid in the cluster to which they were assigned by the end of the clustering process. General practices in a cluster with a higher medoid were classified as having ‘high’ input, while those in a cluster with a lower medoid were classified as having ‘low’ input. Each medoid involved multiple values (from the different variables that defined input), with the sum of these values representing its size. Similarly, K-medoids clustering was applied to the output variables in the linked data to classify general practices as having ‘high’ or ‘low’ output.

K-medoids clustering is an unsupervised machine learning technique that is used to partition datapoints within a dataset into ‘k’ clusters based on their similarity (mathematical closeness). K-medoids clustering was chosen over other methods because it is robust to outliers and does not rely on statistical assumptions about data distribution [23].

For each of the two clustering tasks (one for input and one for output), the following points applied. K-medoids clustering was automated using the ‘kmedoids’ function of the ‘pyclustering’ package (version 0.10.1.2) in Python (version 3.11.7). The data were standardised using z-score normalisation to ensure that clustering was not influenced by differences in the scale of variables. The initial two medoids were randomly selected to minimise the risk of selection bias. A ‘k’ of 2 was used because the aim was to classify each general practice into one of two groups: high or low. For instance, in the input-related clustering task, each general practice was classified into a high-input group or a low-input group. Similarity was assessed using the Manhattan distance, due to its robustness to outliers. All other parameters were left at their default values – ‘tolerance’: 0.0001, ‘itermax’: 200, ‘ccore’: True, and ‘data_type’: ‘points’.

Following the classification of general practices by their input and output levels, efficient, inefficient, and neutral general practices were identified. An efficient general practice was one with a low input and a high output, while an inefficient general practice was one with a high input and a low output. A neutral general practice was one with either both high input and high output or both low input and low output. Neutral general practices were not relevant to addressing the second objective, so they were excluded right after the efficiency determination step.

### Analyses

Summary statistics were computed. Multivariable logistic regression analysis – using the ‘glm’ function in R (version 4.3.3) – was undertaken to identify factors associated with general practice efficiency (i.e. efficient or inefficient). To navigate some logistic regression assumptions, K-medoids clustering was used to convert numerical factors to categorical factors with two categories (high and low). Without clustering, such categorisation would require actual benchmarks – which usually do not exist.

## Results

The linked data initially included 6230 general practices (nearly all the general practices in England in 2023), of which 5069 were included and 1161 were excluded (based on the previously mentioned exclusion criteria). As shown in Table 4, of the 5069, 1117 were classified as efficient, 2773 as neutral, and 1179 as inefficient, based on their input and output levels. The 2773 neutral general practices were excluded from further analyses. Supplementary Tables 1 and 2 provide summary statistics for the variables that defined input and output, respectively.

**Table 4.** Distribution of English general practices by efficiency status in 2023 (*n* = 5069).

Efficient	Neutral	Inefficient
1117 (22%)	2773 (55%)	1179 (23%)

The following numerical factors were converted to categorical factors: ethnicity (white, black, mixed, Asian, and other), number of patients, chronic conditions, deprivation score, and patients 65 years or older. Supplementary Table 3 provides summary statistics for these factors, while Supplementary Table 4 shows the ranges of data representing ‘high’ and ‘low’ in the resulting categorical factors.

Table 5 presents the results of the analyses to identify factors associated with general practice efficiency. Efficiency was lower in general practices with a larger patient list (adjusted odds ratio [aOR] 0.23, 95% CI 0.19–0.28), those with a higher percentage of patients with a chronic condition (aOR 0.47, 95% CI 0.38–0.58) or patients ≥ 65 years (aOR 0.63, 95% CI 0.49–0.81), those in a higher deprivation area (aOR 0.25, 95% CI 0.20–0.32), those that dispense medications (aOR 0.52, 95% CI 0.37–0.73), and those with an alternative provider medical services (vs. general medical services) contract (aOR 0.15, 95% CI 0.07–0.33).

**Table 5.** Results of analyses to identify factors associated with general practice efficiency in 2023 (*n* = 2296).

Factor		Categories within factor	Proportion of efficient general practices in each category (%)	Adjusted odds ratio (95% CI) for each category vs. the reference category	*P*-value for each category vs. the reference category
Number of patients		High	292/906 (32)	0.23 (0.19–0.28)	< 0.001
		Low*	825/1390 (59)	–	–
Rurality		Urban	979/1970 (50)	1.38 (1.00–1.90)	0.05
		Rural*	138/326 (42)	–	–
Deprivation score		High	340/924 (37)	0.25 (0.20–0.32)	< 0.001
		Low*	777/1372 (57)	–	–
Dispensing		Yes	99/296 (33)	0.52 (0.37–0.73)	< 0.001
		No*	1018/2000 (51)	–	–
Contract type		APMS	9/47 (19)	0.15 (0.07–0.33)	< 0.001
		PMS	299/589 (51)	0.99 (0.79–1.23)	0.90
		GMS*	809/1660 (49)	–	–
Chronic conditions		High	424/1193 (36)	0.47 (0.38–0.58)	< 0.001
		Low*	693/1103 (63)	–	–
Patients 65 years or older		High	546/1317 (41)	0.63 (0.49–0.81)	< 0.001
		Low*	571/979 (58)	–	–
Ethnicity	White	High	746/1709 (44)	1.30 (0.79–2.12)	0.30
		Low*	371/587 (63)	–	–
	Mixed	High	530/812 (65)	1.66 (1.24–2.21)	0.001
		Low*	587/1484 (40)	–	–
	Asian	High	275/457 (60)	0.72 (0.49–1.04)	0.08
		Low*	842/1839 (46)	–	–
	Black	High	305/474 (64)	1.31 (0.89–1.92)	0.17
		Low*	812/1822 (45)	–	–
	Other	High	352/509 (69)	1.78 (1.22–2.60)	0.003
		Low*	765/1787 (43)	–	–

Reference category is indicated by an asterisk (*).

APMS: Alternative Provider Medical Services; GMS: General Medical Services; PMS: Personal Medical Services.

In England, GMS, PMS, and APMS are the three types of contract between the NHS and general practices. Among other things, each contract specifies the primary care services that can be provided by general practices that hold it. GMS, the standard contract, is held by most general practices, and it enables them to provide core services. PMS is held by some general practices, and it allows them to provide core services. PMS makes it possible to tailor services to local needs. APMS is held by very few general practices, and it permits them to provide core or non-core services.

Efficiency was higher in urban general practices (aOR 1.38, 95% CI 1.00–1.90) and general practices with a higher percentage of mixed (aOR 1.66, 95% CI 1.24–2.21) or other ethnicity patients (aOR 1.78, 95% CI 1.22–2.60).

## Discussion

### Main findings

We identified factors associated with English general practice efficiency, defining efficiency using a quality-adjusted output.

In this study, general practices with more patients appeared to be less efficient. This finding may be surprising at first glance, but it is not at all surprising when we consider that efficiency was defined using a quality-adjusted output. General practices with more patients generally address a higher population need, and this likely makes it difficult for them to achieve high output (such as good patient experience) even with a seemingly high input. This likely difficulty in the face of a high population need will henceforth be referred to as the high need challenge (HNC) for brevity. General practices with a higher percentage of patients with a chronic condition were less efficient, and general practices with a higher percentage of patients 65 years or older were less efficient. These findings are expected as general practices with a higher percentage of these patients face HNC because these patients generally have poorer health [24]. General practices in higher deprivation areas were less efficient. This is unsurprising as general practices in higher deprivation areas face HNC because patients in these areas generally have poorer health [25]. Dispensing general practices were less efficient than non-dispensing ones. This could be because dispensing general practices employ additional staff to support dispensing, leading to their having an unusually high input. APMS contract general practices were less efficient than GMS ones. General practices with an APMS contract often serve populations, such as homeless individuals or asylum seekers, that typically have high health needs [26,27]. This means they face HNC.

Urban general practices were more efficient than rural ones. General practices in rural areas tend to have a relatively high input (precisely more staff per patient) [28]. It could be that this additional input does not affect output meaningfully, leading to lower efficiency. General practices with a higher percentage of mixed patients were more efficient. Similarly, general practices with a higher percentage of other ethnicity patients were more efficient. An accurate explanation of these ethnicity findings would require critical analysis of the characteristics of these ethnic groups and may benefit from expert insight.

### Comparison with existing primary care literature

The findings of this study align with several studies on primary care efficiency. Bates et al. [12] reported that dispensing general practices were less efficient. Martin and Smith [11] found that primary care trusts in higher deprivation areas were less efficient. Similarly, Takundwa et al. [9] found that clinical commissioning groups serving populations with high unemployment – an indicator of deprivation – were less efficient. Williams et al. [10] and Takundwa et al. [9] found that clinical commissioning groups serving populations with low disease prevalence were more efficient. Likewise, Szczepura et al. [13] found that single-site general practices serving a high percentage of children under five were less efficient, possibly because these general practices face HNC [29]. Takundwa et al. [9] also found that clinical commissioning groups serving large populations were less efficient. Comparably, Giuffrida [8] found that smaller family health services authorities were more efficient.

Some contradictory findings exist. Williams et al. [10] found that clinical commissioning groups with a higher percentage of patients 65 years or older and those in higher deprivation areas were more efficient. Additionally, Szczepura et al. [13] found that larger, multi-site general practices were more efficient. This could be because the authors did not use a quality-adjusted output. Instead, they defined output solely in terms of volume of services. Considering this definition, the contradiction in their findings is unsurprising: such a definition more readily permits primary care practices that face HNC to appear efficient because they typically provide a higher volume of services.

What is evident is that the findings of studies tend to align with ours when the output included clinical quality and/or patient experience, but sometimes differ in other situations. This suggests that it is important to agree on an output definition that we should be using in the primary care efficiency context. At a minimum, output should include quality of care measures. However, we argue for a quality-adjusted output because performance with respect to a combination of throughput and quality of care is arguably more important than performance with respect to either alone.

The choice of input is not as critical as the choice of output because the dimensions of input represent resource capacity and hence often increase (or decrease) together. Therefore, it is not surprising that Neri et al. [22] urge healthcare efficiency assessors to pay close attention specifically to their definition of output.

### Strengths and limitations

#### Strengths

To the best of our knowledge, this is the first study to do the following: (a) determine efficiency using K-medoids clustering, and (b) identify factors associated with primary care efficiency in England using a quality-adjusted output. K-medoids clustering helps efficiently navigate challenges with outliers and varied data distribution, which are typical of datasets used in efficiency determination.

#### Limitations

There is no consensus on which variables best define healthcare efficiency [22], so the efficiency definition in this study may not capture all relevant aspects of general practice performance. However, we believe our definition is relatively appropriate, at least because of the comprehensive nature of our output. Some input variables may be more important than others. However, we did not assign weights to them because it is unclear which ones are more important. The same goes for output variables. The classes to which general practices were assigned using K-medoids clustering are relative and not absolute. The exclusion of some general practices may have affected our findings and reduced their generalisability. Imputation could have prevented exclusions that were specifically due to missing or invalid data. However, the percentage of general practices with missing or invalid data was modest (approximately 6%), making it unlikely that their exclusion was problematic.

### Implications for policy and research

Smaller general practices and general practices with younger or healthier patients were more efficient according to our definition of general practice efficiency. Given that smaller general practices were more efficient, policymakers should not assume that actions, such as general practice mergers, which typically generate economies of scale, will necessarily improve general practice efficiency, at least not efficiency defined using an output that includes quality of care measures.

While comparing our study with the existing literature, we found that the definition of output is associated with the impact of different factors on primary care efficiency. A quality-adjusted output – i.e. an output that accounts for both throughput and quality of care – is arguably better than an output that accounts for either alone. As such, we encourage researchers and policymakers involved in primary care efficiency assessment to adopt such an output.

## Conclusion

There is a need to improve general practice efficiency to help close the gap between demand and supply. In our study, smaller general practices were more efficient. Policy assumptions that increasingly larger general practices will deliver equally increasing efficiency gains may not hold true, and further research is needed to understand the optimal general practice size.

## Supplementary Material

Supplemental Material
