# Supplementary material for: Variation in the efficiency of English general practices and associated factors: A cross-sectional study of 5069 general practices
Source: Eur J Gen Pract. 2025 Nov 17;31(1):2580827. doi: 10.1080/13814788.2025.2580827 (PMC12624881; doi:10.1080/13814788.2025.2580827)
Supplement: Supplemental Material [file IGEN_A_2580827_SM2626.docx]

Supplementary Table 1: Summary statistics for the input variables used to determine the efficiency of general practices (n = 5069).

|  | Minimum | First quartile | Median | Mean | Third quartile | Maximum |
| --- | --- | --- | --- | --- | --- | --- |
| General practice funding in £ (per 10,000 patients) | 18690 | 1139393 | 1303475 | 1462952 | 1582465 | 14913692 |
| GP number (per 10,000 patients) | 1.0 | 3.4 | 4.4 | 4.4 | 5.4 | 9.9 |
| Nurse number (per 10,000 patients) | 0.5 | 1.6 | 2.4 | 2.6 | 3.5 | 9.8 |
| DPC staff number (per 10,000 patients) | 0.0 | 1.0 | 2.0 | 2.4 | 3.3 | 10.0 |
| Administrative staff number (per 10,000 patients) | 0.4 | 9.5 | 11.7 | 12.0 | 14.1 | 29.9 |

DPC, Direct Patient Care; GP, General Practitioner.

Supplementary Table 2: Summary statistics for the output variables used to determine the efficiency of general practices (n = 5069).

|  | Minimum | First quartile | Median | Mean | Third quartile | Maximum |
| --- | --- | --- | --- | --- | --- | --- |
| Continuity (in percent) | 0.0 | 20.0 | 30.0 | 40.0 | 50.0 | 100.0 |
| Access (in percent) | 10.0 | 50.0 | 60.0 | 60.0 | 70.0 | 100.0 |
| Confidence (in percent) | 60.0 | 90.0 | 90.0 | 90.0 | 100.0 | 100.0 |
| Overall satisfaction (in percent) | 20.0 | 60.0 | 70.0 | 70.0 | 80.0 | 100.0 |
| QOF regarding hypertension (in percent) | 0.0 | 10.9 | 14.0 | 12.1 | 14.0 | 14.0 |
| QOF regarding diabetes (in percent) | 0.0 | 11.0 | 14.2 | 13.5 | 17.0 | 17.0 |
| Cancer detection (in percent) | 0.0 | 48.8 | 55.3 | 55.0 | 61.5 | 100.0 |
| General practice appointments (per 10,000 patients) | 1091 | 3357 | 3990 | 4136 | 4751 | 11583 |

QOF, Quality and Outcomes Framework.

QOF is a system used in the UK to incentivise general practices to provide high-quality care to patients. The QOF system has indicators (e.g., QOF regarding hypertension), and general practices are awarded QOF points based on how well they perform against the QOF indicators. *QOF regarding hypertension* represents the percentage of a general practice’s hypertensive patients who are 79 years or younger with a reading of 140/90 mmHg or less in their last blood pressure measurement taken in the preceding 12 months. *QOF regarding diabetes* represents the percentage of a general practice’s diabetic patients – without severe or even moderate frailty – with a reading of 58 mmol/mol or less in their last IFCC-HbA1c test done in the preceding 12 months.

Supplementary Table 3: Summary statistics for the numerical factors (essentially general practice-level demographic data) that were converted to categorical factors (n = 2296).

|  | | Minimum | First quartile | Median | Mean | Third quartile | Maximum |
| --- | --- | --- | --- | --- | --- | --- | --- |
| Ethnicity (in percent) | White | 10.6 | 74.3 | 90.2 | 82.6 | 95.5 | 98.8 |
|  | Black | 0.0 | 0.6 | 1.4 | 3.7 | 4.3 | 38.9 |
|  | Mixed | 0.5 | 1.5 | 2.4 | 2.9 | 3.9 | 9.1 |
|  | Asian | 0.4 | 1.7 | 4.5 | 8.8 | 11.4 | 70.8 |
|  | Other | 0.1 | 0.5 | 1.0 | 2.1 | 2.7 | 21.5 |
| Number of patients  (raw value) | | 1291 | 6659 | 9674 | 11316 | 13564 | 86861 |
| Chronic conditions  (in percent) | | 19.5 | 49.5 | 55.8 | 55.5 | 61.8 | 81.4 |
| Deprivation score  (raw value) | | 3.4 | 13.2 | 20.2 | 22.4 | 29.6 | 65.2 |
| Patients 65 years or older  (in percent) | | 0.0 | 14.0 | 18.7 | 18.3 | 22.4 | 42.1 |

Supplementary Table 4: Ranges of data representing ‘high’ and ‘low’ in the categorical factors derived – using K-medoids clustering – from the numerical factors summarized in Supplementary Table 3 (n = 2296).

|  | | High | Low |
| --- | --- | --- | --- |
| Ethnicity (in percent) | White | 75.0-98.8 | 10.6-74.8 |
|  | Black | 5.6-38.9 | 0.0-5.6 |
|  | Mixed | 3.1-9.1 | 0.5-3.1 |
|  | Asian | 13.6-70.8 | 0.4-13.6 |
|  | Other | 3.0-21.5 | 0.1-3.0 |
| Number of patients (raw value) | | 11173-86861 | 1291-11133 |
| Chronic conditions (in percent) | | 55.4-81.4 | 19.5-55.4 |
| Deprivation score (raw value) | | 23.3-65.2 | 3.4-23.2 |
| Patients 65 years or older (in percent) | | 17.5-42.1 | 0.0-17.5 |
